# Information-Theoretical Quantifier of Brain Rhythm Based on Data-Driven Multiscale Representation

**DOI:** 10.1155/2015/830926

**Published:** 2015-08-24

**Authors:** Young-Seok Choi

**Affiliations:** ^1^Department of Electronic Engineering, Gangneung-Wonju National University, Gangneung 210-702, Republic of Korea; ^2^Research Institute for Dental Engineering, Gangneung-Wonju National University, Gangneung 210-702, Republic of Korea

## Abstract

This paper presents a data-driven multiscale entropy measure to reveal the scale dependent information quantity of electroencephalogram (EEG) recordings. This work is motivated by the previous observations on the nonlinear and nonstationary nature of EEG over multiple time scales. Here, a new framework of entropy measures considering changing dynamics over multiple oscillatory scales is presented. First, to deal with nonstationarity over multiple scales, EEG recording is decomposed by applying the empirical mode decomposition (EMD) which is known to be effective for extracting the constituent narrowband components without a predetermined basis. Following calculation of Renyi entropy of the probability distributions of the intrinsic mode functions extracted by EMD leads to a data-driven multiscale Renyi entropy. To validate the performance of the proposed entropy measure, actual EEG recordings from rats (*n* = 9) experiencing 7 min cardiac arrest followed by resuscitation were analyzed. Simulation and experimental results demonstrate that the use of the multiscale Renyi entropy leads to better discriminative capability of the injury levels and improved correlations with the neurological deficit evaluation after 72 hours after cardiac arrest, thus suggesting an effective diagnostic and prognostic tool.

## 1. Introduction

Electroencephalogram (EEG) has been exploited in connection with functional brain mechanisms as a potential tool for the identification of brain disorder such as hypoxic-ischemic brain injury and epileptic seizure [1, 2]. Despite the effectiveness of EEG as a clinical diagnostic tool, most interpretations are based on subjective measures such as visual inspection, limiting precise interpretation. Thus, the need for objective measures gives rise to the development of quantitative EEG measure to uncover neurological states. Recently, quantitative EEG analyses based on novel signal processing techniques have shown promising results for deriving quantitative patterns that may correspond to diagnostic information and cognitive deficits [3–8].

Among those, information theoretic analyses such as entropy measure have been successfully used to quantify the degree of irregularity of injured brain rhythm [9–12]. These studies founded on assumption that the larger the information content of EEG, the better the neurological status of brain. More recently, it has been reported that informative content in EEG spans and varies over multiple frequencies through injury and recovery phases [11, 13, 14]. Thus the single scale based entropy measures are lacking in reflecting the changing dynamics over multiple scales in EEG.

To address this obstacle, this paper presents a multiscale based entropy measure by incorporating the empirical mode decomposition (EMD) method into computing entropy. The EMD method, which has been recently introduced as a data-driven and adaptive technique, is known to be appropriate for analyzing nonstationary and nonlinear time-series [15]. It decomposes a time-series into a number of narrowband components, called intrinsic mode functions (IMFs), by empirically identifying the physical time scales intrinsic to the signal. Thus, due to the potential of EMD, it has been gradually used to analyze neurophysiological recordings such as EEG [16–18] and field potential [19, 20]. In addition, since it is known that EMD behaves as a dyadic filter bank [21, 22], it is well fit for detecting the dynamics of the frequency bands of interest in EEG study [17]. Upon the results of EMD of EEG, the proposed measure computes Renyi entropy [23] using the probability distributions of IMFs at each scale, followed by averaging Renyi entropies over multiple scales. Thus, the resultant multiscale Renyi entropy reflects distinct features over multiple scales which are derived from a data-driven way.

To demonstrate the performance of the proposed multiscale Renyi entropy, simulation and experimental studies using a synthetic signal and an animal model during brain injury and recovery after cardiac arrest have been carried out. The performance of the multiscale Renyi entropy was demonstrated by comparing with the conventional single scale one in terms of both how well it discriminates the degree of uncertainty and predicts neurological outcomes.

The remainder of the paper is organized as follows.  Section 2 provides a brief description on EMD and the multiscale Renyi entropy measures. Sections 3 and 4 validate the proposed approach via simulation and experimental studies.  Section 4 presents the discussion and conclusion.

## 2. Materials and Methods

### 2.1. Empirical Mode Decomposition

This section summarizes a data-driven decomposition method, that is, EMD, which has been developed by Huang et al. in 1998 [15]. The EMD method is a novel signal processing method which represents a time-series into a finite set of amplitude and frequency modulated oscillating components which are bases of the decomposition scheme. The decomposition procedure of EMD is an adaptive signal-dependent technique. In an iterative manner, termed a sifting process, EMD extracts the highest frequency oscillation (finest temporal scale) from the underlying time-series, referred to as an intrinsic mode function. The remaining part after the extraction contains lower frequency oscillatory components. The resultant IMFs represent the oscillatory patterns over multiple scales. This gives rise to the following major feature of EMD: EMD results in basis functions which are derived from the time-series in self-originated way, whereas other conventional methods such as Fourier and wavelet analyses rely on the use of predefined basis functions.

An IMF has to meet the following two criteria: (1) the number of extreme and zero crossings are either equal or differ by at most one and (2) the mean value of the envelope defined by the local maxima and local minima is zero.

Here, we describe the principle of EMD as follows. Let *s*(*i*) denote the raw sampled EEG signal. Then EMD method consists of the following steps.

(1) Identify all the local maxima and minima of *s*(*i*).
(2) Interpolate between local maxima and minima, respectively, getting an upper envelope *e*_*u*_(*i*) and a lower envelope *e*_*l*_(*i*).
(3) Compute the mean between *e*_*u*_(*i*) and *e*_*l*_(*i*); that is, *μ*(*i*) = [*e*_*u*_(*i*) + *e*_*l*_(*i*)]/2.
(4) Subtract the mean from the original signal
  (1) $$\begin{array}{c} d\left(i\right)=s\left(i\right)-\mu \left(i\right). \end{array}$$
(5) Repeat steps (1)–(4) until *d*(*i*) satisfies the above two criteria to be an IMF. If *d*(*i*) satisfies conditions, it becomes the first intrinsic mode function that contains the finest temporal scale in the signal. Also it is denoted by *d*_1_(*i*).
(6) Compute the residue *r*_1_(*i*) = *s*(*i*) − *d*_1_(*i*).
(7) Iterate through steps (1)–(6) with *r*_1_(*i*) instead of *s*(*i*) until the residue satisfies some stopping criterion as
  (2) $$\begin{array}{c} \text{SD}=\frac{\sum_{}{\left|d\left(i\right)-s\left(i\right)\right|}^{2}}{\sum_{}s^{2}\left(i\right)}<\alpha , \end{array}$$
 where *α* is an arbitrary value in the range of 0.2–0.3 as recommended in [15].

Through the sifting process, the raw EEG signal *s*(*i*) is decomposed as follows:

(3) $$\begin{array}{c} s\left(i\right)=\overset{K}{\underset{k=1}{\sum }}d_{k}\left(i\right)+r_{K}\left(i\right), \end{array}$$

where *K* is the number of all extracted intrinsic mode functions, *d*_*k*_(*i*) is the *k*th intrinsic mode function, and *r*_*K*_(*i*) is the final residue. The last residue *r*_*K*_(*i*) can be considered as the last IMF and thus 3 can be rewritten as

(4) $$\begin{array}{c} s\left(i\right)=\overset{K+1}{\underset{k=1}{\sum }}d_{k}\left(i\right). \end{array}$$

In addition, from 3, it is obvious that EMD is complete; that is, *s*(*i*) can be reconstructed from the resulting IMFs and the final residue. Also it is known that the resulting IMFs are nearly orthogonal; thus they can be considered as the basis to represent the underlying time-series [15, 21, 22].

### 2.2. Computing Time-Dependent Multiscale Entropy

In the proposed method, we utilize the distribution of the time-varying individual oscillatory components, that is, *d*_*k*_(*i*), obtained in 3 in evaluating the multiscale Renyi entropy. To cope with the temporal evolution of entropy, EEG recording is divided into a number of segments using a sliding temporal window, leading to a time-dependent entropy measure [24]. For a given {*s*(*i*) : *i* = 1,…, *N*}, a sliding temporal window *w* ≤ *N* and a sliding interval Δ ≤ *w* are defined. Then, the *n*th sliding window of the raw EEG signal is defined by

(5) $$\begin{array}{c} s_{n}\left(i\right)=\left\{s\left(i\right):i=1+n\Delta ,\ldots ,w+n\Delta \right\}, \end{array}$$

where *n* = 0,1,…, [(*N* − *w* + 1)/Δ], and [*x*] denotes the integer part of *x*.

Then, we incorporate EMD to utilize the underlying time-varying oscillatory components in EEG recording. Assume that EEG is decomposed into IMFs by a sifting process, yielding totally *K* IMFs and one residual which is considered as (*K* + 1)th oscillatory component. A set of IMFs is obtained from the EEG signal in a sliding window **s**_*n*_(*i*), which is given by

(6) $$\begin{array}{c} EMD\left[s_{n}\left(i\right)\right]=\left[d_{n}^{1},d_{n}^{2},\ldots ,d_{n}^{K+1}\right], \end{array}$$

where **d**_*n*_^*k*^ = [*d*_*k*_(*i*) : *i* = 1 + *n*Δ,…, *w* + *n*Δ] for *k* = 1,…, *K* + 1 are the *k*th IMF after EMD on the *n*th sliding window.

In order to compute the probability distributions of the IMFs, **d**_*n*_^*k*^ is partitioned into *M* disjoint intervals {*I*_*m*_, *m* = 1,…, *M*} spanning the range between the minimum and maximum IMF with *l*_*l*_ = min⁡{**d**_*n*_^*k*^} and *l*_*M*_ = max⁡{**d**_*n*_^*k*^} where *l*_*l*_ < *l*_2_ < ⋯<*l*_*M*_. Using the above definitions, a set of disjoint intervals {*I*_*m*_ = [*l*_*m*_, *l*_*m*+1_], *m* = 1,…, *M* − 1} is obtained by binning **d**_*n*_^*k*^. Next, *p*_*n*_^*k*^(*m*) is the probability that the IMF belongs to the interval *I*_*m*_ in *k*th IMF **d**_*n*_^*k*^. It is computed as a ratio of number of samples of **d**_*n*_^*k*^ within *I*_*m*_ and the total sample number of **d**_*n*_^*k*^.

To evaluate multiscale based Renyi entropy, the probabilities of each IMF are incorporated into well-known Renyi entropy as follows.

(1) For each intrinsic mode function, the Renyi entropy (RE) in each intrinsic mode is calculated as

(7) $$\begin{array}{c} \text{R}{\text{E}}^{k}\left(n\right)=-\frac{1}{q-1}\ln\left(\overset{M}{\underset{m=1}{\sum }}{\left(p_{n}^{k}\left(m\right)\right)}^{q}\right), \end{array}$$

where *k* = 1,…, *K* + 1, 0 ≤ *p*_*n*_^*k*^(*m*) ≤ 1, and ∑_*m*=1_^*M*^ *p*_*n*_^*k*^(*m*) = 1.

(2) The following averaged Renyi entropies over all scales lead to the multiscale Renyi entropy (MRE) as follows:

(8) $$\begin{array}{c} \text{MRE}\left(n\right)=\overset{K+1}{\underset{k=1}{\sum }}\text{R}{\text{E}}^{k}\left(n\right). \end{array}$$

**Figure 1:**
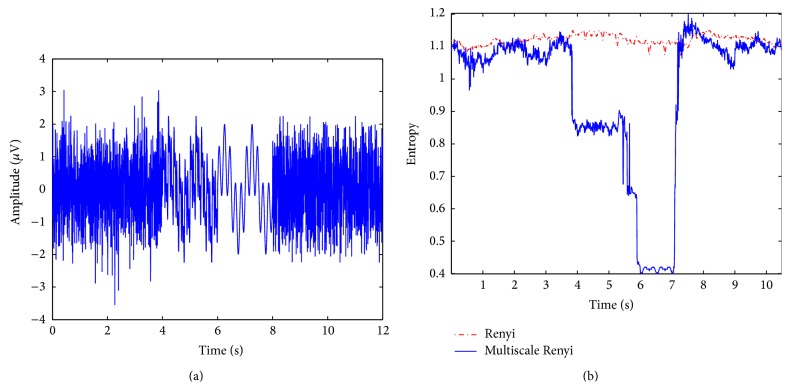
Time evolution of the conventional Renyi entropy and multiscale Renyi entropy for synthetic signal with time-varying frequency components. (a) Synthetic signal in time domain. (b) Comparison of Renyi entropy and multiscale Renyi entropy.

To compare the multiscale Renyi entropy with the single scale based one, that is, Renyi entropy of gross EEG, computer simulation was carried out. A synthesized signal consisting of Gaussian distribution and multiple sinusoidal components was used, which is shown in Figure 1(a). The sampling frequency for the synthetic signal was 256 Hz. For the first 4 s, the synthetic signal has Gaussian distribution. Following period of the synthetic signal has different number of sinusoids in time-dependent manner as follows. From 4 s to 6 s, it consists of 4 sinusoids whose frequencies are at 1, 5, 10, and 20 Hz. From 6 s to 8 s, it is composed of 2 sinusoids with 1 and 5 Hz. During last 4 s, the random permutation surrogate of the period between 4 s and 8 s was included.  Figure 1(b) depicts the results of the conventional Renyi entropy and the proposed multiscale Renyi entropy, respectively. As can be seen, two entropy measures show similar levels for Gaussian distribution. From 4 to 8 s, the conventional Renyi entropy is almost constant regardless of the number of sinusoids, whereas the multiscale Renyi entropy decreases in accordance with the decrease of sinusoids. During last 4 s, the multiscale Renyi entropy increased, having comparable level of the conventional one.

### 2.3. Animal Model and EEG Recordings

EEG signals were recorded from rats during experiments in rodents subjected to controlled periods of normal circulation and asphyxial cardiac arrest with the goal of assessing brain dynamics following such an injury. The experimental model of brain injury by cardiac arrest has been approved by Animal Care and Use Committee of the Johns Hopkins Medical Institutions. This rat model has been previously validated to study multiple aspects of calibrated brain injury after asphyxial cardiac arrest, including the physiologic parameters, short term and long term neurobehavioral outcomes, EEG recovery, and histology [25, 26].

Nine adult male Wistar rats (300 ± 25 g) were used. Anesthesia was induced with 4% halothane in 50% N_2_ : 50% O_2_. 10 min of baseline trend was recorded including 5 min washout period to ensure that halothane did not influence the EEG. Subsequently, 7 min asphyxia was induced by stopping and disconnecting the ventilator and clamping the tracheal tube. The duration of cardiac arrest was determined by the mean arterial blood pressure being below 10 mmHg. The cardiopulmonary resuscitation (CPR) was carried out by chest compression until return of spontaneous circulation (ROSC), which was defined as mean arterial blood pressure (MABP) higher than 60 mmHg. Selected rats received hypothermia therapy. The therapy involved cooling the core body temperature to 32–34°C through surface cooling with misted water immediately (within 15 min) after return of spontaneous circulation and therapeutic hypothermia was maintained for 6 hours. Then, the rats were gradually rewarmed to 37°C for 2 hours. Four rats under normothermia (37°C) and others under immediate hypothermia (32–34°C) were selected; EEG signals were recorded using two channels from the right and left parietal regions of rat's brain using subdermal needle electrodes (Plastics One, Roanoke, VA). ECG and arterial pressure were also recorded simultaneously.

**Figure 2:**
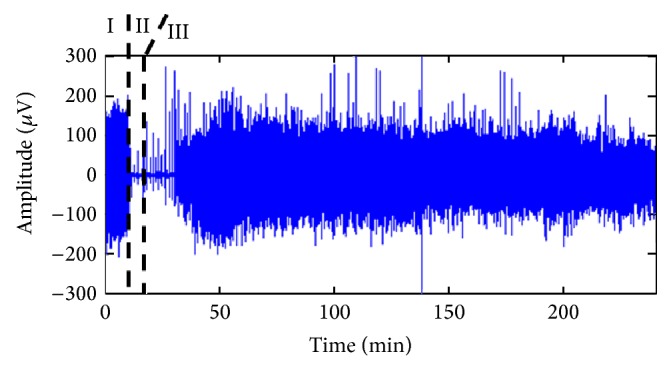
Raw EEG recording of a rat during brain injury and recovery after asphyxia cardiac arrest. A 4-hour compressed signal capturing the entire experiment is presented. (I) 10 min baseline, (II) 7 min brain injury after cardiac arrest and silent period, and (III) EEG recovery.

The signals were digitalized using CODAS, a data acquisition package (DATAQ Instruments INC., Akron, OH). A sampling rate of 250 Hz and a 12-bit resolution of A/D converter were used for digitization of the data. All rats were resuscitated and neurological outcome was evaluated by neurological deficit score (ranging from 0 = worst to 80 = best) consisting of level of arousal, cranial nerves and sensory motor assessments, reflexes, and occurrence of clinically appreciable seizures [27]. The neurological deficit score was calculated by an independent observer 72 h after asphyxial cardiac arrest injury.  Figure 2 shows the time trend of the experiment, with Phase I being the control period, Phase II the global ischemic brain injury, and Phase III the recovery period.

## 3. Results

Figure 2 shows the EEG recording for a rat during brain injury and recovery after cardiac arrest. The raw EEG signal can be divided into three periods as follows: (I) 10 min baseline, (II) 7 min CA and silent phase, and (III) recovery. From Figure 2, it is obvious that the amplitude of EEG decreases after CA injury and is followed by gradual increase in recovery period. However, it is difficult to clearly discriminate difference between the preinjury and the various recovery phases by visualization alone. Even more difficulty would be to objectively compare different injury grades or the effects of hypothermia therapy. Limits of visual investigation stress the need for a reliable quantitative approach to study EEGs.

**Figure 3:**
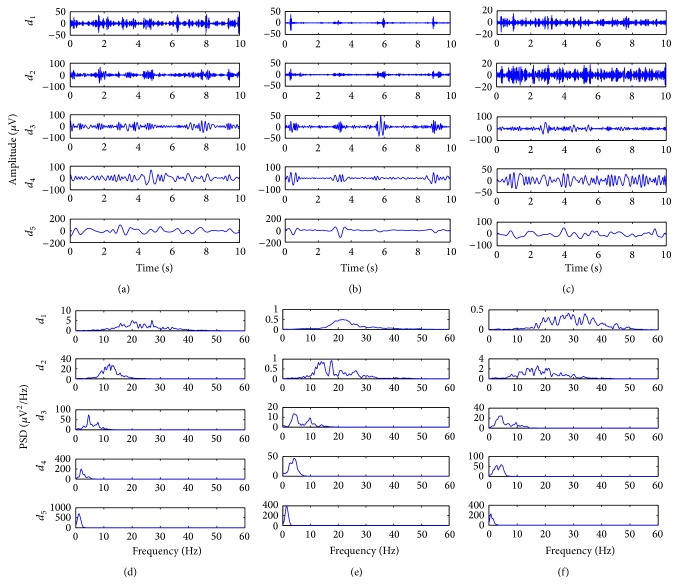
The EMD results of real EEG recording. For three phases of the experiment, that is, (a) baseline, (b) early recovery, and (c) late recovery, the time domain representations of the resulting IMFs of the 10 s segments of EEG are shown (top: highest scale (*d*_1_), bottom: lowest scale (*d*_5_)). (d)–(f) The power spectral densities corresponding to (a)–(c), respectively.

To show the inherent oscillatory components of EEG, that is, IMFs, the EMD method was carried out, and the resulting IMFs and corresponding power spectral densities are shown in Figure 3. Figures 3(a)–3(c) show the EMD results of three 10 s segments of EEG recording at various phases in Figure 1 as follows: EEG recordings in baseline, 50 min, and 180 min, respectively. With the power spectral density shown in Figures 3(d)–3(f), we could observe that each intrinsic mode function approximates the clinical bands of EEG; that is, the first IMF covers *γ* and *β* bands (>16 Hz), and the second and third IMFs show *α* and *θ* bands (4–16 Hz). As expected, the resulting IMFs over multiple scales cover the clinical band of interest while maintaining a good decorrelation property.

For evaluating the multiscale complexities, the following parameters were used: sliding temporal window length with *w* = 10 s, sliding interval with Δ = 10 s and *M* = 20. In addition, when computing Renyi entropies, we choose *q* = 3 as suggested in [28]. The resulting multiscale Renyi entropy values were averaged across left and right brain areas for each rat. In addition, the entropy measures were normalized with respect to average values over baseline period (0–10 min).

**Figure 4:**
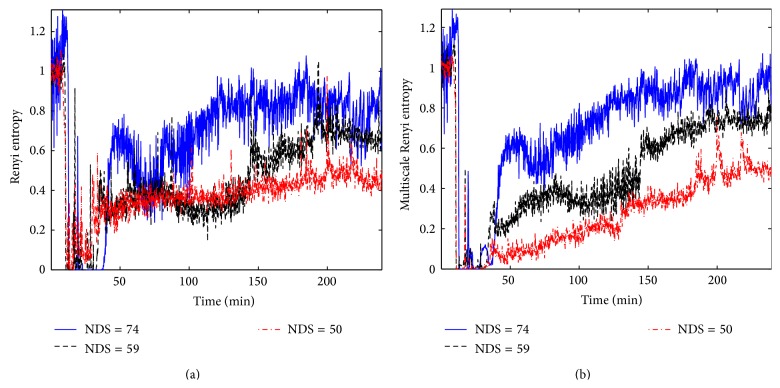
Time evolutions of the conventional Renyi and multiscale Renyi entropies for three rats (NDS = 74, 59, and 50). (a) The Renyi entropies. (b) The multiscale Renyi entropies (*q* = 3).

Figure 4 shows the time evolutions of the conventional Renyi entropy and the multiscale Renyi entropy for 3 rats with eventual good, medium, and poor outcomes (neurological deficit score (NDS) = 74, 59, and 50 on a scale of 0 (worst) to 80 (best)). Figures 4(a) and 4(b) illustrate the results of the Renyi entropies and the multiscale Renyi entropies of three rats, respectively. In both plots, after washout around 15 min, entropies of 3 rats dramatically fall to approximately zero. Occurrence of a spike at 22 min was due to manual resuscitation. The Renyi entropies in Figure 4(a) rapidly increase from 35 to 40 min. Renyi entropy values during recovery (Figure 4(a)) are not highly separable for different animals with different neurological deficit scores. On the other hand, the multiscale Renyi entropies in Figure 4(b) for the 3 rats are consistently separable for those with different neurological deficit scores. These results indicate that the higher the neurological score, the higher the entropy value at the end of the 4-hour recovery period.

To assess above results with a larger sample, the Renyi and multiscale Renyi entropies of 9 rats including the previous 3 rats were calculated as shown in Table 1. Here, aggregate data for each rat is organized into rows, arranged, and numbered in order of increasing of the neurological deficit score. The results of Renyi and multiscale Renyi entropies are presented together, with Renyi entropy results enclosed in parentheses. To demonstrate the entire trend, entropies for each rat were averaged over selected intervals and the average of recovery phase (30–240 min from the start of experiment). To analyze the capability of entropies as a predictor of neuronal recovery, we evaluate Pearson correlation coefficient and hypothesis testing using *P* value between neurological deficit score and entropies over the selected intervals and the whole recovery period recorded (30–240 min). From Table 1, Pearson correlation coefficients between the multiscale Renyi entropy and neurological deficit score were more significant over all given time slots than between Reni entropy and neurological deficit score. Additional hypothesis testing using a Student-t distribution (*n* = 9) was conducted. The results of hypothesis testing also support that the multiscale Renyi entropy is more correlated with neurological deficit score than the multiscale Renyi entropy's counterpart, revealing the improved predictability of multiscale Renyi entropy for discerning neurological status.

## 4. Discussion and Conclusion

This work presents a new framework for quantifying information quantity in EEG over multiple time scales. Entropy has been considered to reflect the underlying dynamics in EEG. Hence, various entropy measures have been successfully applied in prognosticating the degree of neurological states. However, most methods which are based on a single scale have limitation in describing information content spanned over different scales. It leads to a need for multiscale based entropy measure to capture locally changing feature at various frequencies or scales.

This analysis of experimental EEG signals has been done in two parts. First, we recognize that the EEG signals, recorded during experimental interventions (such as global ischemia brain injury reported here), are inevitably nonstationary. In addition, their composition is complex, with different modes or basis components, constituting the EEG rhythm at any time instant. Therefore, a data-driven analysis method, namely, EMD, was utilized to decompose the EEG signal at different time instants during the experimental investigations. Second, the analysis yields that EEG signals demonstrate considerable entropy which varies during different experimental stages. Here, in order to quantify the information quantity of EEG over adaptive and data-dependent multiple scales, Renyi entropy of IMFs which are results of EMD has been incorporated. As the precedent approaches, the entropy-based EEG analysis methods for hypoxic-ischemic injury have shown their capability for evaluating recovery from brain injury [24, 28–30]. In [24, 28], the quantification of entropy depended on a gross EEG recording, which may lose the multiscale dynamics. Also, the studies in [29, 30] developed multiple frequency band analysis based on wavelet transform, which is not sufficient for representing nonstationary neural data. Comparing to the previous approaches, this work yields a multiscale analysis based on EMD, which makes it suitable for analyzing neural data.

Along this line, some multiscale entropy works have been presented in [31, 32]. In [31], an adaptive multiscale entropy along each scale based on multivariate EMD (MEMD) has shown promising capability for representing dynamics of neural data. In addition, Hu and Liang [32] have used an additional noise channel to identify information-bearing components in neural data, resulting in a noise-robust analysis approach.

Through simulation and experimental studies, the results demonstrated that the multiscale Renyi entropy measures show a stronger correlation with the clinical measure of the neurological deficit score than conventional single scale based Renyi entropy. For asphyxial cardiac arrest model of rat, the results show that the proposed multiscale Renyi entropy leads to high correlation with the eventual neurological deficit score at each phase of the EEG recording. As a future step, the integration with the approaches in [31, 32] would increase the efficacy as a quantitative neurological measure for clinical EEG studies.

To conclude, a novel multiscale Renyi entropy framework for analysis of EEG signals has been presented. Analysis of experimental EEG recording has shown that multiscale Renyi entropy correlates well with clinically relevant measures of neurological deficits. This study lays the foundation for applying this novel approach to clinical studies of human EEG signals recorded during comparable episodes of brain injury resulting from global ischemia after cardiac arrest as well as other clinical situations such as traumatic brain injury.

## Figures and Tables

**Table 1:** Statistical results of Renyi and multiscale Renyi entropies.

Rat ID	Multiscale Renyi entropy (Renyi entropy)				NDS
	30–60 min	60–120 min	120–240 min	Ave.	
#1	0.24 (0.51)	0.33 (0.54)	0.50 (0.55)	0.41 (0.54)	46
#2	0.07 (0.30)	0.17 (0.36)	0.40 (0.44)	0.28 (0.40)	50
#3	0.24 (0.29)	0.36 (0.35)	0.63 (0.58)	0.50 (0.47)	59
#4	0.65 (0.75)	0.80 (0.85)	0.78 (0.81)	0.76 (0.82)	74
#5	0.43 (0.40)	0.65 (0.58)	0.88 (0.83)	0.74 (0.69)	74
#6	0.44 (0.56)	0.54 (0.59)	0.76 (0.77)	0.65 (0.68)	74
#7	0.54 (0.60)	0.63 (0.84)	0.65 (0.60)	0.63 (0.67)	75
#8	0.42 (0.52)	0.63 (0.68)	0.74 (0.80)	0.66 (0.72)	78
#9	0.40 (0.50)	0.60 (0.68)	0.61 (0.63)	0.58 (0.63)	80
*r*	0.79 (0.49)	0.86 (0.69)	0.75 (0.75)	0.84 (0.76)	
*P*	0.01 (0.18)	0.003 (0.04)	0.02 (0.02)	0.004 (0.02)	

*r*: correlation coefficient, *P*: *P* value, and NDS: neurological deficit score.
